# Examining the Dimensions of the Bicipital Groove: A Human Cadaveric Study

**DOI:** 10.7759/cureus.50895

**Published:** 2023-12-21

**Authors:** Anthony N Baumann, Grayson M Talaski, Andrew Fiorentino, Katelyn Sidloski, Hudson Rogers, Caleb J Oleson, Tobin L Hieronymus, Albert T Anastasio, John Martin Leland

**Affiliations:** 1 Department of Rehabilitation Services, University Hospitals, Cleveland, USA; 2 Department of Orthopedics, University of Iowa, Iowa City, USA; 3 College of Medicine, Northeast Ohio Medical University, Rootstown, USA; 4 Department of Orthopedic Surgery, Duke University, Durham, USA; 5 Department of Orthopedic Surgery, University Hospitals, Cleveland, USA

**Keywords:** musculoskeletal, cadaver, biceps anatomy, biceps pathology, biceps groove

## Abstract

Introduction

Understanding the mechanisms and risk factors associated with bicipital groove (BG) morphology is essential for optimizing patient outcomes. Despite interest in the topic of BG morphology, there remains a lack of clarity and consensus on the parameters of BG morphology due to significant methodological limitations in the existing research. The purpose of this study is to explore the dimensions of BG morphology with a methodology rooted in recent research findings to better understand the human anatomy, potentially underpinning various shoulder pathologies.

Methods

The right shoulders of seventeen cadavers (nine male and eight female; median age of death: 88.0 years; age of death range: 66.0 - 97.0 years) were included in this cadaveric study. Dissection was done by removing the deltoid musculature and reflecting the long head of the biceps tendon to expose the BG. Measurements for BG morphology included BG width (millimeters, mm), depth (mm), and length (mm). Statistical comparisons were done between male and female measurements using the independent-samples Mann-Whitney U test due to the small sample size.

Results

The median width of the BG at the narrowest point was 4.3 mm (mean: 4.7 ± 1.4 mm; range: 3.0 - 7.7 mm) with male cadavers having a significantly wider BG as compared to female cadavers (median: 5.0 mm versus 3.7 mm; p=0.006). The median depth of the BG was 5.1 mm (mean: 5.0 ± 0.7 mm; range: 3.8 - 6.3 mm) with no statistically significant difference between male and female cadavers (median: 4.8 mm versus 5.3 mm; p=0.370). The median length of the BG was 25.1 mm (mean: 25.1 ± 3.3 mm; range: 18.1 - 31.3 mm) with no statistically significant difference between male and female cadavers (median: 25.4 mm versus 23.9 mm; p=0.673).

Conclusion

The width of the BG at the narrowest point was significantly larger in male cadavers as compared to female cadavers in this study. However, there was no difference between male and female cadavers in terms of depth and length of the BG. This study contributes to the understanding of BG morphology by exploring the dimensions for width, depth, and length of the BG, which may contribute to biceps tendon pathology in clinical practice. Future research should focus on reducing measurement variability and exploring the possible relationship between BG morphology and biceps tendon conditions to further enhance the understanding of this complex relationship.

## Introduction

The bicipital groove (BG), a bony sulcus between the greater and lesser tubercles of the proximal humerus, allows for the smooth transition of the long head of the biceps tendon as it extends from the supraglenoid tubercle of the scapula, through the joint capsule of the shoulder, and eventually into union with the short head of the biceps tendon [1]. As damage to the long head of the biceps tendon can lead to significant pain and changes in function, understanding the mechanisms and risk factors behind poor biceps stabilization in the BG is relevant for clinical practice [2]. While most tendon excursion occurs at the level of the soft tissue, studies have shown equal importance for a properly contoured BG in aiding the proper motion mechanics of the biceps tendon [3-5]. The shallow depth and abnormal bony contours of the BG, such as a bone spur, have been correlated to long-head bicep instability and pathology, suggesting that while proper soft-tissue involvement is crucial for biceps stabilization, the morphology of the BG may further portend symptomatology and need for intervention [4,5].

Numerous studies have analyzed the BG with respect to biceps pathology, but results have yet to reach a common consensus [6-9]. However, there are major flaws in studies that analyze BG morphology, or attempt to correlate morphology to a risk factor, due to several methodologic errors [4,10]. One major error is that these studies compare BG morphology across patient populations that have bicep injuries, which could be a significant confounding factor [4,10]. Without a potentially healthy reference for BG morphology, it may be difficult to understand the impact of BG morphology on possible instability and patient symptoms. Assessing this relationship in an injured group does not account for the method of injury and patient type (active vs. nonactive before injury), which may be major risk factors for instability. Furthermore, previous attempts to assess the healthy morphology of the BG did not account for sex differences, thus limiting application for male or female patients [11]. Furthermore, methodological flaws were also noted in these studies in terms of measurement strategies [4,10,11]. For example, one study only measured width at the midpoint, which may not be the best indicator of width as the narrowest part of the BG could possibly have the greatest impact on the biceps tendon [4]. Therefore, further investigation is warranted in which the width of the BG is measured at the narrowest point. Over the past decade, much has been learned about the risk factors of biceps tendon pathology and this study will be able to draw upon these results when assessing BG morphology in a human cadaver population.

Therefore, the purpose of this study is to explore the dimensions of the BG (depth, length, and width) to guide surgeon decision-making and patient education on the possible etiology and risk factors for biceps pathology. The results of this study can allow for future studies examining biceps pathology to determine which dimensions of the BG, if any, serve as a risk factor for biceps tendinitis, biceps tendinosis, and tear/rupture.

## Materials and methods

Cadaver demographics

A total of 20 human cadavers from an anatomy lab were available for examination in this study. After three cadavers were excluded due to significant osteoarthritis resulting in severe disfigurement of the BG (found on dissection), a total of 17 cadavers were included in this study. There were nine male cadavers and eight female cadavers, and the right shoulder was examined for each cadaver. Refer to Table 1 for individual cadaver demographics. The median age of death for the entire cohort (n=17 cadavers) was 88.0 years (mean: 84.2 ± 9.0 years; range: 66.0 - 97.0 years). Male cadavers (n=9 cadavers) had a median age of death of 88.0 years (mean: 84.3 ± 8.4 years; range: 66.0 - 92.0 years) and female cadavers (n=8 cadavers) had a median age of death of 84.5 years (mean: 84.1 ± 10.2 years; range: 71.0 - 97.0 years). This study is considered exempt as cadaver research is not considered human research based on current ethical definitions. 

**Table 1:** Individual cadaver demographics and biceps groove measurements for this study. Data recorded includes cadaver number (1-17), age of death (years), sex (male/female), biceps groove width (millimeters, mm), biceps groove length (mm), and biceps groove depth (mm).

Cadaver number	Age of death (years)	Sex	Biceps groove width (mm)	Biceps groove length (mm)	Biceps groove depth (mm)
1	90	Male	4.9	25.4	4.0
2	94	Female	3.2	18.1	5.3
3	82	Male	7.7	25.5	4.6
4	92	Female	3.0	23.6	5.6
5	79	Male	4.1	29.6	5.2
6	97	Female	3.9	22.7	4.2
7	66	Male	4.3	24.3	4.7
8	90	Male	6.2	27.6	5.8
9	74	Female	4.3	24.2	3.8
10	91	Male	7.3	25.1	3.9
11	71	Female	5.1	31.3	5.2
12	88	Male	4.3	25.7	5.7
13	79	Female	3.4	26.0	4.9
14	92	Male	6.3	22.4	4.8
15	76	Female	4.2	22.4	5.9
16	81	Male	5.0	22.6	5.1
17	90	Female	3.4	30.4	6.3

Dissection methods

**Figure 1:**
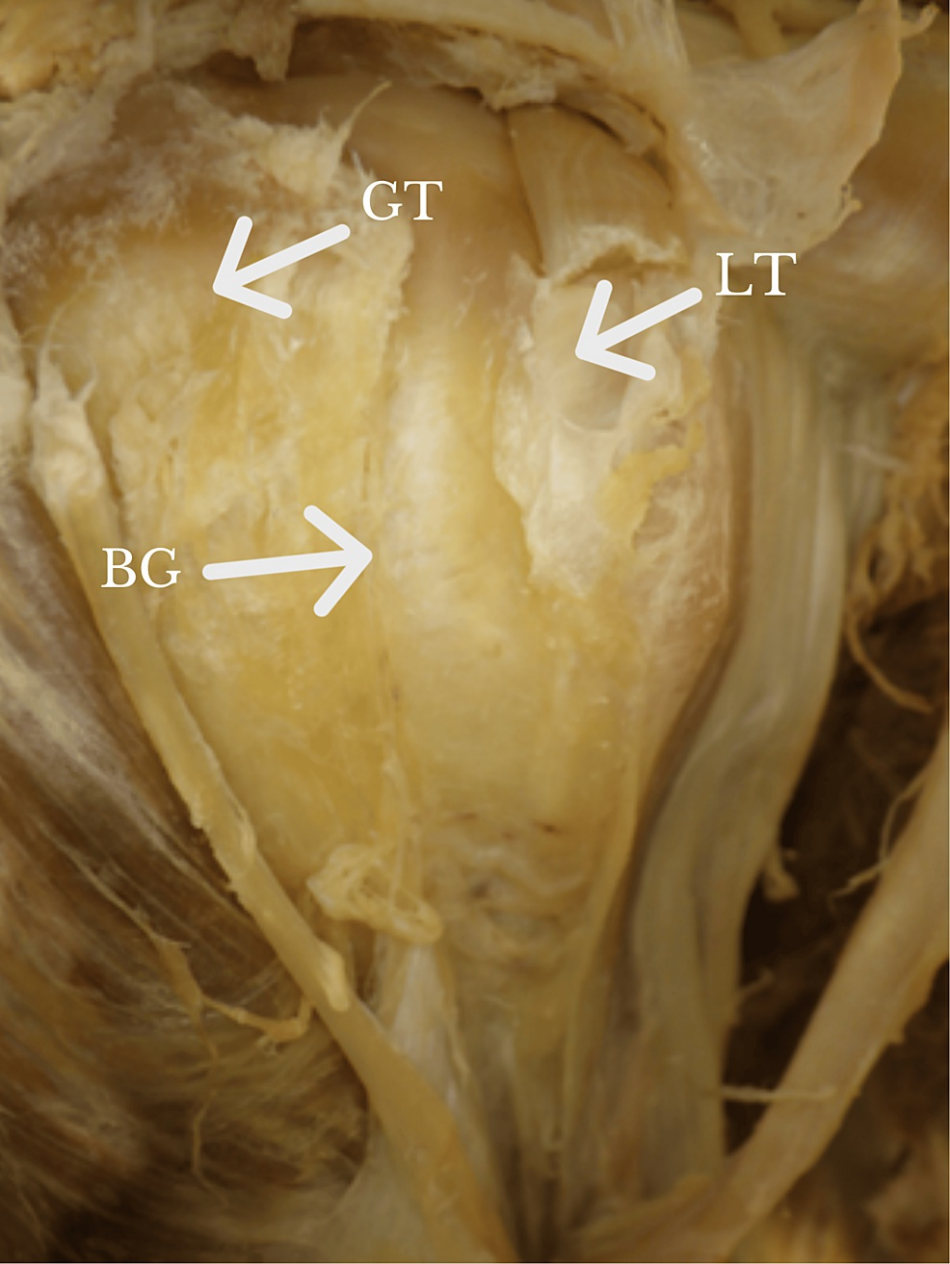
Image of a BG with the biceps tendon resected. BG: bicipital groove; GT: greater tuberosity; LT: lesser tuberosity Orientation: The left side of the figure is the lateral, whereas the right side of the figure is the medial.

All cadavers available to the authors in an anatomy lab were included as long as they had no significant osteoarthritis in their right shoulder upon dissection. All cadavers had the skin and adipose tissue removed from the right shoulder region prior to this study for teaching purposes of the anatomy lab in which this study was conducted. With regard to cadaver preparation for this investigation, the first step of dissection was to elevate the deltoid muscle from the clavicle and resect the lateral deltoid musculature to expose the anterior shoulder and the long head of the biceps. Once any surrounding fascia and adipose tissue were removed so that the long head of the biceps was clearly visible, a probe was placed under the long head of the biceps to free it from the underlying BG via rupture of the transverse humeral ligament. Then, the long head of the biceps tendon was cut superior to the plane of the greater and lesser tubercle and then resected inferiorly. Once the long head of the biceps tendon was resected inferiorly, this allowed visualization and measurement of the BG. Multiple authors assisted in the dissection of the cadavers for this study after being shown the proper dissection methodology. Refer to Figure 1 for an image of the fully dissected and prepped BG.

Measurement definitions

All measurements were taken by a single author who teaches as a PhD in the cadaver lab. The measurements were taken with Mitutoyo calipers (Model number: 505.672; Mitutoyo Corporation, Kawasaki, Kanagawa, Japan) in millimeters (mm). The width of the BG was measured as the narrowest portion of the BG when approaching from superior to inferior. The length of the BG had a superior boundary and an inferior boundary with the measurement consisting of the distance between the two boundaries. The superior boundary was the start of the palpable lateral border of the lesser tubercle of the humerus and the inferior boundary was the end of the palpable lateral border of the lesser tubercle of the humerus. The depth of the BG was measured at the qualitatively deepest part of the BG on visual inspection. Data recording was completed by the first author.

Statistical analysis

The IBM SPSS Statistics for Windows, Version 29, (Released 2022; IBM Corp., Armonk, New York, United States) was utilized for this study. Measures of central tendency and descriptive statistics such as mean, standard deviation, mean, and range were used to describe the data. For statistical comparison between BG measurements (length, width, and depth of the BG in mm) and sex of the cadaver (male, female), the independent-sample Mann-Whitney U test was used due to the small sample size. The significance for this study was set at 0.05.

## Results

BG width

**Figure 2:**
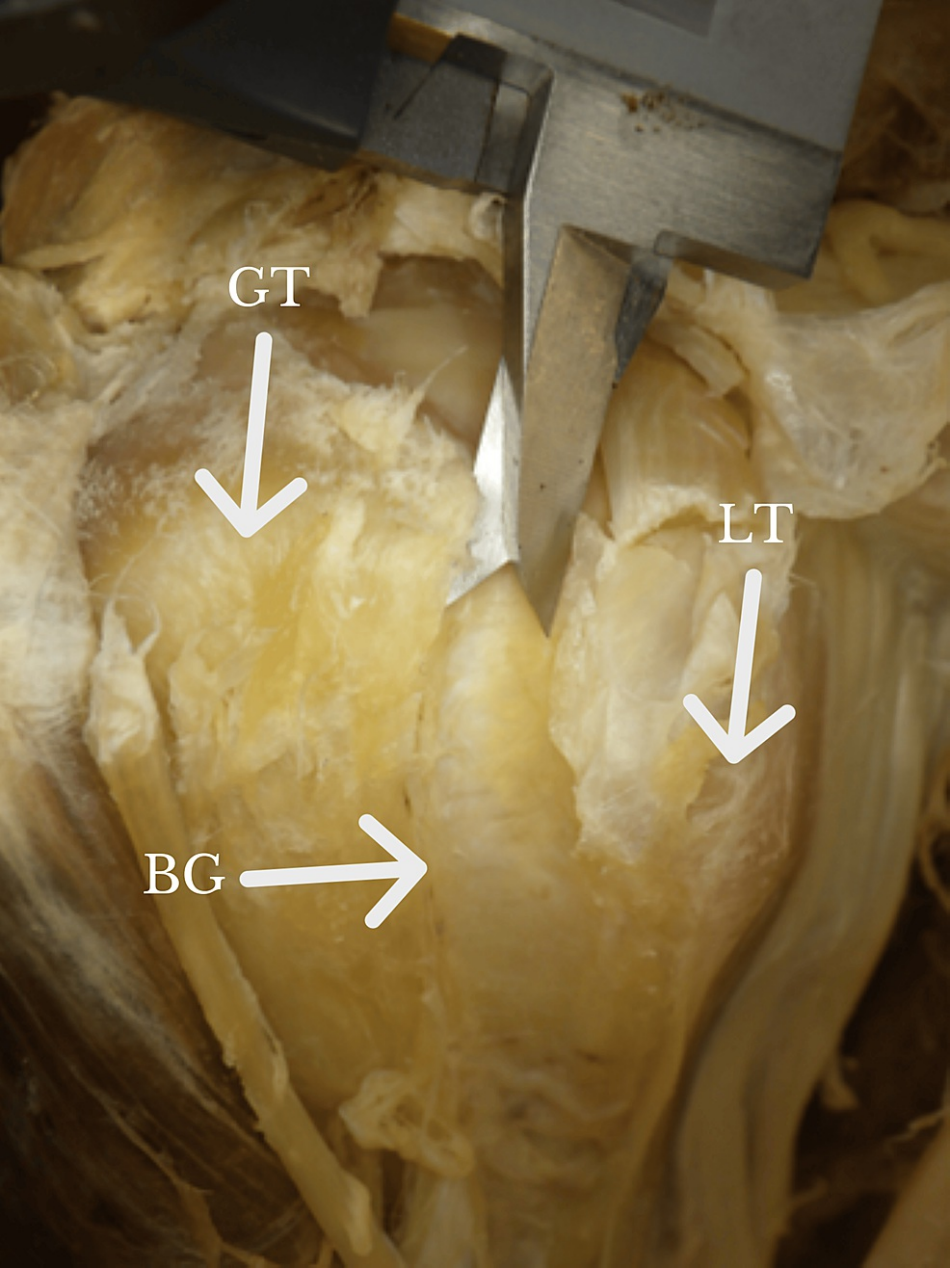
Calipers measuring the width of the BG at the narrowest portion. BG: bicipital groove; GT: greater tuberosity; LT: lesser tuberosity Orientation: The left side of the figure is the lateral, whereas the right side of the figure is the medial.

The median BG width for the entire cohort (n=17 cadavers) was 4.3 mm (mean: 4.7 ± 1.4 mm; range: 3.0 - 7.7 mm). Refer to Table 1 for individual cadaver width of the BG measurements and Table 2 for the average cadaver width of the BG. There was a statistically significant difference in the average BG width based on the sex of the cadaver with male cadavers having significantly greater BG width than female cadavers (median: 5.0 mm versus 3.7 mm; p=0.006). Refer to Figure 2 for an image of the measurement of the width of the BG using the calipers in this study.

**Table 2:** Average age of death and biceps groove measurements for the cadavers in this study by the entire cohort, male cadavers, and female cadavers. Measurements were reported with mean ± standard deviation, median, and range. mm: millimeters; SD: standard deviation Significance was marked by an asterisk (*).

Measurements	Group	Mean ± SD	Median	Range	p-values
Age of death (years)	Entire cohort (n=17)	84.2 ± 9.0	88.0	66.0 - 97.0	-
	Male (n=9)	84.3 ± 8.4	88.0	66.0 - 92.0	
	Female (n=8)	84.1 ± 10.2	84.5	71.0-97.0	
Biceps groove width (mm)	Entire cohort (n=17)	4.7 ± 1.4	4.3	3.0 - 7.7	-
	Male (n=9)	5.6 ± 1.4	5.0	4.1 - 7.7	p=0.006*
	Female (n=8)	3.8 ± 0.7	3.7	3.0 - 5.1	
Biceps groove length (mm)	Entire cohort (n=17)	25.1 ± 3.3	25.1	18.1 - 31.3	-
	Male (n=9)	25.4 ± 2.3	25.4	22.4 - 29.6	p=0.673
	Female (n=8)	24.8 ± 4.3	23.9	18.1 ± 31.3	
Biceps groove depth (mm)	Entire cohort (n=17)	5.0 ± 0.7	5.1	3.8 - 6.3	-
	Male (n=9)	4.9 ± 0.7	4.8	3.9 - 5.8	p=0.370
	Female (n=8)	5.2 ± 0.8	5.3	3.8 - 6.3	

BG depth

**Figure 3:**
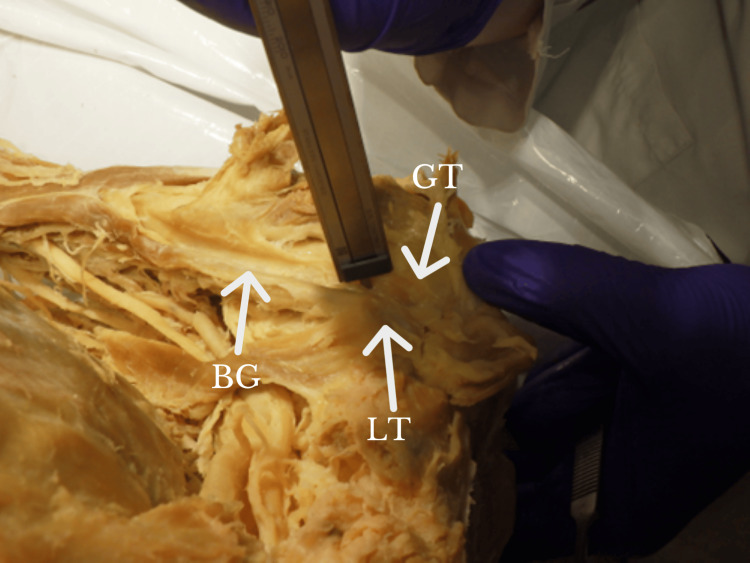
Calipers measuring the depth of the BG. BG: bicipital groove; GT: greater tuberosity; LT: lesser tuberosity Orientation: The left side of the figure is the lateral, whereas the right side of the figure is the medial.

The median BG depth for the entire cohort (n=17 cadavers) was 5.1 mm (mean: 5.0 ± 0.7 mm; range: 3.8 - 6.3 mm). Refer to Table 1 for individual cadaver depth of the BG measurements and Table 2 for the average cadaver depth of the BG. There was no statistically significant difference in the average BG depth based on the sex of the cadaver (median: 4.8 mm versus 5.3 mm; p=0.370). Refer to Figure 3 for an image of the measurement of the depth of the BG using the calipers in the study.

BG length

**Figure 4:**
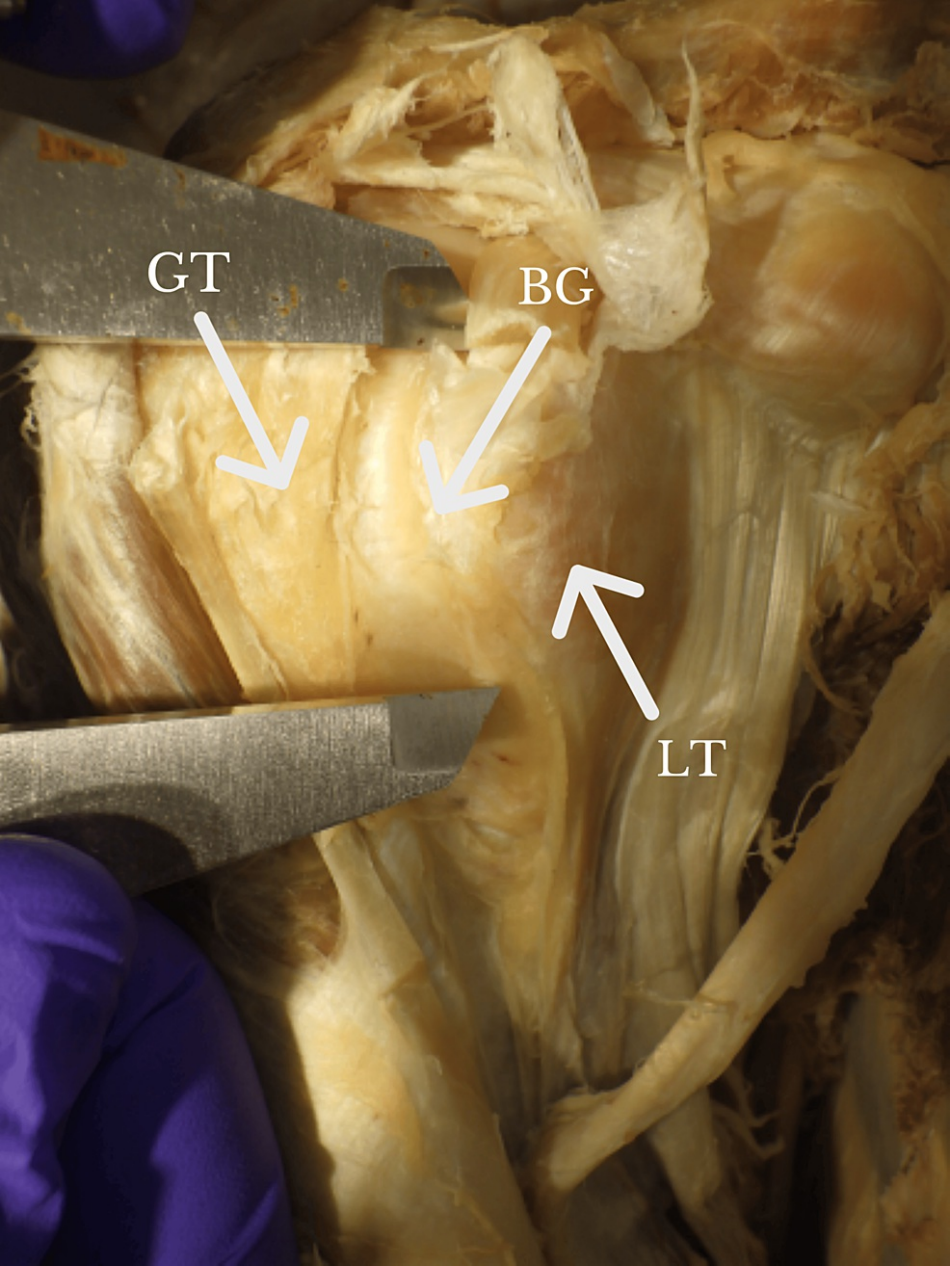
Calipers measuring the length of the BG. BG: bicipital groove; GT: greater tuberosity; LT: lesser tuberosity Orientation: The left side of the figure is the lateral, whereas the right side of the figure is the medial.

The median BG length for the entire cohort (n=17 cadavers) was 25.1 mm (mean: 25.1 ± 3.3 mm; range: 18.1 - 31.3 mm). Refer to Table 1 for individual cadaver length of the BG measurements and Table 2 for the average cadaver length of the BG. There was no statistically significant difference in the average BG length based on the sex of the cadaver (median: 25.4 mm versus 23.9 mm; p=0.673). Refer to Figure 4 for an image of the measurement of length of the BG using the calipers in the study.

## Discussion

In this cadaveric study, the dimensions of the BG were measured and analyzed to provide a clearer picture of BG morphology. As studies have suggested the impact that BG morphology could possibly have on biceps stability, further exploration of the BG via a cadaveric study with good methodology was of high priority at this time [4,5]. While the literature indicates that BG morphology has little impact on stability, it is possible that prior study methodology may have prevented proper analysis of this relationship [10]. In a study that directly evaluated the stability of the long-head bicep tendon, the investigative group underwent shoulder arthroscopy for other pathologic conditions, indicating a possible flaw in methodology [10]. This flaw in methodology is a confounding variable that prevents true analysis as patients with biceps pathology may have different BG morphology as compared to healthy patients. As studies have shown a possible genetic link for the proclivity of developing various shoulder injuries, such as rotator cuff tears, analyzing the BG morphology of patients that may be predisposed to biceps instability is not a reliable approach [12]. This flawed methodology of using shoulders with confirmed pathology and/or pain is common across nearly all studies that found no relationship between BG morphology and ligamentous instability [6].

However, it is important to note that evidence highlighting the importance of investigating BG morphology is abundant [4,5,13-15]. While many of these studies also followed a similar flawed methodology of studies that found little impact of BG morphology on instability, the results of this study can be interpreted as being more reliable due to the cadaver population as opposed to a population with confirmed shoulder pathology [4,5,10,11]. For example, in a study that found a significant impact of BG width, depth, and curvature on detecting injury, the studied populations had confirmed ligamentous disorders [5]. Overall, more research is needed to determine the impact of BG morphology on not only the presence of biceps pathology but the severity of biceps pathology as well. 

Recent evidence has suggested that BG depth, width (often reflective of opening angle), and bony disruptions such as bone spurs can contribute to shoulder pathology [4,5,13]. With this knowledge, it becomes crucial to accurately reflect the normative values of these regions. Previous attempts to measure the width of the BG returned much higher values than the values in this present study, with our average width being nearly 50% lower than previous studies [11,16]. This difference can be attributed to the methodology of our study, which is a direct result of the increase in knowledge of the impact of the BG on biceps instability. Previous studies measured width at the BG midpoint, which may not be the best indicator of width [11,16]. In this present study, the width was reflective of the narrowest point of the BG. While no study has directly studied the impact of the width of the narrowest point, narrower opening angles of the BG have been linked to a lower prevalence of biceps instability in injured patients [4]. We argue that the width of the narrowest part of the BG is a type of measure of the opening angle of the narrowest portion of the BG. Furthermore, previous attempts to explore the dimensions of the BG failed to appropriately establish sex-related differences in BG morphology [11]. In the present study, we found significant differences between male and female cadavers regarding BG width. Again, as width has been a proven indicator of ligamentous instability, using proper methodology to differentiate between males and females when defining healthy width values is crucial [4,5,13]. Using evidence from the long timeframe that separates our study from previous attempts is a large advantage that will allow for the values presented herein to have true meaning in a clinical setting.

While accurately defining the BG was important, the main priority of this study was to explore aspects of BG morphology that could potentially be used to predict biceps tendon pathologies and clinical outcomes in future studies. Having healthy values for BG morphology to reference may help clinicians when deciding upon treatment options. As certain labor-intensive occupations can lead to increased stress on the long head of the biceps tendon, the morphology of the BG would be expected to widen and decrease in depth with the increased pressures from the larger muscle [17,18]. Using these reference values, a physician could determine a patient’s deviation from normal and possibly offer insight into methods to strengthen supplementary muscles that may limit the risk of biceps instability. Depth in this study was measured in a cadaveric model, which may be difficult to replicate, but other studies have found a similar depth reading on traditional radiographs [18]. While a cadaveric model is a limitation in that replication is difficult, future studies can reference the values in this study when attempting to measure BG morphology using imaging modalities as hands-on measurements are directly reflective of the true BG morphology. Furthermore, our study demonstrated a high variability in BG measurements. Future research should aim to develop measurements that focus on the important aspects of the bicep groove, such as width and depth while establishing lower variability with a larger sample size that allows for a clearer distinction of those at higher risk of injury.

It is important to acknowledge the limitations of this study. First, our study had a relatively small sample size. However, this is the largest sample size to date that successfully demonstrates differences in sex for BG morphology while also including measurements that have shown clinical significance. Next, some measurements could be considered more subjective than others and each measurement was only taken by a single author, thus allowing for possible error in measurement. Additionally, no clear landmark existed when assessing length, which made consistent measurement difficult. However, as no study has shown the importance of BG length, this discrepancy has little impact on predicting and treating bicep pathologies. Next, it was impossible to determine if the specimens included had bicep tendon pathologies which may have undermined our methodology. It is unknown if the cadavers had experienced shoulder pain, shoulder pathology, or shoulder surgery in the past, which introduces a confounding variable into our data. Future research comparing a cadaver cohort with confirmed healthy shoulders to those with confirmed bicep pathologies may strengthen the results of this study. Furthermore, this study did not measure the biceps tendon condition. As the tendon can often change shape when there is pathology, pain, or patient complaint, the morphology of the BG may likewise undergo modifications. Future research into the impact of bicep tendon morphology with changes in bicep condition may demonstrate if this measurement has any true significance. Next, angular measurements were not taken. As these measurements have shown significance regarding ligamentous instability in recent studies, future research should include these metrics when defining normality values. As angular measurements can be difficult to take in a consistent manner on a cadaveric model, this study did not attempt to include angular metrics. Finally, measurements were only performed by one author, which did not allow for interobserver comparison.

## Conclusions

The width of the BG at the narrowest point was statistically significantly larger in male cadavers as compared to female cadavers in this study, indicating possible differences in BG morphology that need to be further explored. However, there was no statistically significant difference between male and female cadavers in terms of depth and length of the BG. This study advances the understanding of BG morphology by emphasizing the critical dimensions of the BG. By offering a measurement of the narrowest part of the BG and recognizing the importance of differences between males and females, this study represents improvement over previous research in the field. These findings hold crucial clinical significance by setting a foundation for future clinical studies that can help guide surgeons when assessing and treating patients with biceps-related conditions. Further research can build upon findings to assess the impact of BG morphology as a risk factor for bicep pathologies and impact patient care. 
